# Within-child associations between sleep quality and emotional self-regulation over 6 months among preschool-aged (3- to 5-year-old) children

**DOI:** 10.3389/frsle.2024.1420245

**Published:** 2024-08-07

**Authors:** Cassandra M. Godzik, Delaina D. Carlson, Oleksandra I. Pashchenko, Grace A. Ballarino, Jennifer A. Emond

**Affiliations:** ^1^Department of Psychiatry, Dartmouth College and Dartmouth-Hitchcock Medical Center, Lebanon, NH, United States; ^2^Department of Psychiatry, Dartmouth College, Hanover, NH, United States; ^3^Department of Epidemiology, Geisel School of Medicine at Dartmouth College, Lebanon, NH, United States; ^4^Department of Family Medicine, Full Circle Health, Boise, ID, United States; ^5^Department of Pediatrics, Geisel School of Medicine at Dartmouth College, Lebanon, NH, United States; ^6^Department of Biomedical Data Science, Geisel School of Medicine at Dartmouth College, Lebanon, NH, United States

**Keywords:** emotional dysregulation, self-regulation, early childhood, sleep problems, sleep quality

## Abstract

**Objective:**

We leveraged an observational, repeated-measures study to examine the within-child associations between sleep quality and emotional self-regulation, controlling for between-child effects.

**Methods:**

Children aged 3–5 years and one parent each were recruited from the community in northern New England between 2019 and 2022. Parents completed online surveys at baseline and 2, 4, and 6 months post-baseline. Child sleep quality was measured with the validated Children's Sleep Habits Questionnaire modified for preschoolers; higher scores indicate worse sleep quality (range 32–96). Child emotional self-regulation was measured with the validated Child Social Behavior Questionnaire; higher scores indicate better emotional self-regulation (range 1–7). Adjusted mixed-effects linear regression was used to model the associations between nighttime sleep quality (exposure) and emotional self-regulation (outcome) measured at each of the four study time points while disaggregating the between- and within-child effects.

**Results:**

Children (*n* = 91) were largely white, non-Hispanic (88.7%), and from a higher social-economic status. Sleep quality scores averaged 38.9 (*SD*: 6.6) at baseline; 23.1% of children had scores >41, which is considered evidence of significant sleep problems. Emotional self-regulation scores averaged 4.2 (*SD*: 1.0). There was a significant within-child association between sleep quality and emotional self-regulation. Specifically, a decrease in sleep quality at any one time point, relative to each child's mean sleep quality, related to worse emotional self-regulation [standardized beta, βs = −0.31; 95% CI (0.53, −0.09)]; the between-child effect was not significant. Results were consistent when limited to children with complete data at all study visits (*n* = 78).

**Conclusions:**

The findings support a causal, within-child association between sleep quality and emotional self-regulation in preschool-aged children, with effects evident over 6 months.

## Introduction

Sleep is an important part of adequate growth, normal cognitive development, and healthy daytime functioning in children (Meltzer et al., 2010; Gruber et al., 2014; Bathory and Tomopoulos, 2017). Average sleep duration recommendations change with age and vary significantly throughout adolescence. For example, the National Sleep Foundation recommends 10–13 h of sleep over 24 h for children aged 3–5 years (Hirshkowitz et al., 2015). However, children often struggle with adequate sleep, particularly in the first 5 years of the child's life as the sleep–wake system continues to develop and the frequency and duration of daytime napping decreases (Meltzer et al., 2010; Gruber et al., 2014; Bathory and Tomopoulos, 2017). From ages 3–5, as many as 50% of preschool-aged children were reported to wake up at least once per night (Gruber et al., 2014). Consistent nighttime wakings can result in poor nighttime sleep quality and, if not addressed, may develop into a sleep disorder that could potentially lead to future health problems (Gruber et al., 2014; Hirshkowitz et al., 2015). Not only is sleep important for maintaining a healthy weight, immune system, and cardiovascular health, but it is also important for good neurocognitive development, mental health, executive functioning, and likely emotional regulation (Gruber et al., 2014; Bathory and Tomopoulos, 2017; Schumacher et al., 2017).

Emotional regulation is generally defined as modulating one's emotional changes in a way that is conducive to the task one is working to complete (Graziano et al., 2007; Braund and Timmons, 2021). Among preschool-aged children, emotional regulation is often framed as the ability to self-regulate behavioral responses to situations that provoke emotions (e.g., anger, sadness, excitement) (Cole et al., 2009; Sala et al., 2014). In contrast, difficulties with emotional regulation at this age may manifest as externalizing behaviors including crying, yelling, and other physical gestures reflecting anger, sadness, or even extreme joy. In particular, early childhood is an important period for executive functioning and emotional regulation development (Schumacher et al., 2017; Braund and Timmons, 2021). Poor emotional regulation in childhood has been linked with behavioral problems and worse academic success in later life (Graziano et al., 2007; Schumacher et al., 2017; Williams et al., 2017; Braund and Timmons, 2021). Furthermore, early childhood sleep problems have been associated with future substance use disorders (i.e., alcohol, nicotine, and illicit drugs) (Gruber et al., 2014). One study that aimed to understand the relationship between adolescent sleep disorders and early substance abuse found that “sleep problems at ages 3–8 predicted onset of alcohol, cigarette, and marijuana use among boys and onset of alcohol use among girls” (Wong et al., 2009). Thus, thoroughly exploring the relationship between inadequate sleep and emotional dysregulation in childhood is important.

Numerous studies have attempted to establish a link between early childhood sleep problems and the onset of emotional and behavioral problems in preschool years. A long-term study by Sivertsen et al. (2015) compared irregular patterns in 18-month-olds to the incidence of emotional and behavioral problems by age 5. A sleep duration of ≤ 10 h combined with frequent nocturnal awakenings at 18 months of age significantly predicted behavioral and emotional dysregulation at age 5 (Sivertsen et al., 2015). Another study by Williams et al. (2017) utilized a longitudinal, cross-lagged approach and reported that sleep problems were significantly related to worse emotional regulation 2 years later when children were followed from infancy to 9 years of age. While these studies support that poor sleep quality at a young age is associated with poorer emotional self-regulation later in childhood, the understanding of shorter term within-child effects of poor sleep on emotional self-regulation is limited. Filling that gap can help inform whether interventions targeting better sleep hygiene at a young age can have a measurable impact on children's emotional self-regulation in the short term.

Traditional statistical models using observational data typically combine the within- and between-child effects. Between-child effects represent how the mean level of exposure relates to the mean level of the outcome. In contrast, within-child effects represent how the outcome (e.g., self-regulation) changes when the exposure (e.g., sleep quality) changes within each child. Between-child effects may be influenced by residual confounding while within-child effects more precisely measure potential causal associations (Curran and Bauer, 2011). Regression models that disaggregate the between- vs. within-child effects are becoming more common in health behavior research because these methods can more precisely address potential causal relationships (Curran and Bauer, 2011; Dunton et al., 2019; Emond et al., 2021; Bonar et al., 2022). For example, a recent study by McAlpine et al. (2024) examined both between- and within-subject factors to determine the causal relationship between sleep hygiene behaviors and nighttime sleep duration. Results from that study indicated that different factors were related to poor sleep duration when considering between-subject (daytime napping, alcohol use, social media use, etc.) vs. within-subject factors (poor sleep conditions, changes in sleep/wake times, etc.) (McAlpine et al., 2024). Findings support that to truly understand factors causally linked to poor sleep, statistical models that isolate the between- and within-subject effects are needed.

The goal of this study was to examine the associations between sleep quality and emotional self-regulation among preschool-aged children, aged 3–5 years, leveraging repeated measures of both sleep and self-regulation to examine within-child changes.

## Methods

### Study site and participants

This is a secondary analysis of data collected from a 6-month prospective, observational study among preschool-aged children designed to assess screen media use and obesogenic eating behaviors. Children aged 3–5 years and one parent each (inclusive of guardians) were recruited from the community from July 2019 through October 2022; study activities took place in Northern New England, United States. Children were ineligible if they had any significant developmental or language delays, sleep disorders, food allergies or dietary restrictions, appetite- or attention-altering disorders, or if they could not comprehend English. Additionally, participants were excluded if they had an immediate family member who had previously participated in the study or if the family was planning to relocate within 6 months. Parents also had to have primary (>50%) custody of their child. Parents provided informed consent, and children provided verbal assent. Dyads visited the lab at baseline and month 6, and parents completed online surveys baseline and 2-, 4-, and 6-months post-baseline. Participants were compensated up to US$170 for completing all study activities. Dartmouth's Committee for the Protection of Human Subjects approved all study protocols. Data for this study were from the parent-reported online surveys at each study time point.

### Children's sleep quality

Parents completed the Children's Sleep Habits Questionnaire (CSHQ) at each study visit. The CSHQ is a validated assessment of sleep quality initially developed for 4- to 11-year-old children (Owens et al., 2000) with documented validity for 2- to 5-year-old children (Goodlin-Jones et al., 2008). The CSHQ includes 33 items to measure sleep quality based on sleep behaviors and characteristics across eight subdomains: bedtime resistance, sleep onset delay, sleep duration, sleep anxiety, night wakings, parasomnias, sleep-disordered breathing, and daytime sleepiness. Parents rate how frequently their child exhibits the behavior for each specific item: *rarely (0–1 time per week), sometimes (2–4 times per week)*, or *usually (5–7 times per week)*; parents were asked to consider the past week when completing the CSHQ for the current study. Item responses are summed to a final score (some items are reverse-coded) such that a higher score reflects poorer sleep quality. Final scores on the original questionnaire range from 33 to 99, with scores >41 considered evidence of clinically significant sleep problems (Owens et al., 2000). In alignment with the modified version of the CSHQ validated for preschool-aged children (Goodlin-Jones et al., 2008), this current analysis did not include the bed-wetting item in the total CSHQ score because bed-wetting may be an age-appropriate behavior for this life stage. Thus, the possible range of our CSHQ score was 32–96. Cronbach's alpha for the CSHQ in the current sample was 0.82.

### Children's sleep quantity

Children's nighttime sleep quantity was parent-reported as part of the CSHQ. Specifically, parents reported how long their child usually sleeps each night, considering the time asleep, not simply time in bed. Responses were prepopulated in a drop-down menu and presented as hours in 30-min increments (e.g., 6, 6.5, 7 h). Parents also reported how often their child usually napped during the day, also reported in 30-min increments. Nighttime sleep and daily naps were summed to compute the usual total 24-h sleep.

### Children's emotional self-regulation

Child emotional self-regulation was assessed with the emotional self-regulation subscale of the Child Social Behavior Questionnaire (CSBQ). This scale was initially developed for children with an autism spectrum disorder or pervasive developmental disorder (Luteijn et al., 1998; Hartman et al., 2006) and has been adopted to measure emotional self-regulation in healthy preschool-aged children (Anderson et al., 2017; Howard and Melhuish, 2017; Forrest et al., 2020). Parents reported how much each of the five items reflected their child's behaviors as related to regulating their mood and temper: *My child…(1) shows wide mood swings, (2) is impulsive and acts without thinking, (3) gets overexcited, (4) gets over being upset quickly*, and *(5) is easily frustrated*. Items were scored on a 7-point Likert scale (one item reverse-scored), with anchors *extremely untrue* (1) to *extremely true* (7), and the final score is the mean across all items (range 1–7). Scores for this analysis were reverse-scored such that a higher score reflected better self-regulation. Cronbach's alpha at baseline for this 5-item scale in the current sample was 0.77.

### Covariates

Potential covariate measures, all measures were parent-reported at the baseline visit, included the following: child age, biological sex, and race/ethnicity; parent educational attainment, marital status, and annual household income; and the number of children < 18 years old living in the home. Parents also reported on the child's total screen media use (watching TV; using a computer, smartphone, and/or tablet; or playing video game consoles) for a weekday and, separately, a weekend day. Values were weighted and summed to create a weekly total for children's screen media use. Parents also completed the 15-item authoritative and the 5-item permissive subscales of the Parenting Styles and Dimensions Questionnaire (PSDQ), a questionnaire that characterizes parents based on their responsiveness and demandingness scored on a 5-point Likert scale (Robinson et al., 1995). An average score was calculated for each parenting style. The Cronbach's alpha of the PSDQ subscales of authoritative and permissive parenting styles for the current sample at baseline were 0.85 and 0.68, respectively. A higher score on authoritative parenting style indicates more nurturing and responsive parenting with consistent, fair discipline; a higher score on permissive parenting style indicates less disciplined and structured parenting with overindulgence to avoid conflict.

In addition, parents completed the 15-item Confusion, Hubbub, and Order Scale (CHAOS) (Matheny et al., 1995) questionnaire to assess household chaos, which the survey defines as hurriedness or disorder in the home. Items are scored on a 4-point Likert scale, from *not at all* (1) to *very much* (4), regarding the parent's home. The final score is a sum of responses (range 15–60) with a higher score reflecting a more chaotic home environment. The CHAOS questionnaire has been validated against direct observations of parental and household behaviors (Matheny et al., 1995). Cronbach's alpha for the chaos scale in the current sample was 0.86. Household chaos was considered a covariate because of existing associations between more household chaos and behavioral problems/poor sleep quality among children (Emond, 2020; Marsh and Dobson, 2020).

### Statistical analyses

Linear mixed-effects regression was used to model the associations between child sleep quality (independent variable) and emotional self-regulation (dependent variable), with emotional regulation included in the model in two ways. First, to compare usual sleep quality between children (i.e., between-subject effect), each child's mean sleep quality score over the 6-month study period (i.e., the mean of score at baseline and each follow-up assessment) was included in the model. Second, for each child, we computed the difference in sleep quality at each time point relative to their mean, and those differences were included in the model to reflect the within-child effect at each study time point. This modeling process addresses how each child's emotional self-regulation changes when they experience better or worse quality sleep relative to their mean sleep quality. The between-child effect further absorbs any residual confounding related to sleep and emotional self-regulation; thus, the within-child effect better approximates possible causality than would be possible with methods that do not disaggregate these two sources of variation. The model included a random effect at the child level to account for the repeated measures over time. Emotional self-regulation and sleep quality scores were standardized to *z*-scores before including in the model to standardize the interpretation of effect sizes, and the model was adjusted for covariates. Covariates were selected a priori (child age, child sex, and study visit time point) and included baseline measures associated with child emotional self-regulation or nighttime sleep quality at baseline at the *p* < 0.10 level in unadjusted analyses (permissive parenting style, child media use). Model residuals were examined, and no violations of model assumptions were apparent.

Two sets of sensitivity analyses were conducted. First, the final model was rerun, limited to the subset of participants with complete data at all four study time points. Second, because the COVID-19 pandemic began during the study enrollment period, the final model was repeated, including an indicator variable to denote if the baseline study visit was before or after the pandemic started (March 11, 2020) (CDC Timeline, n.d.). This was to account for children who started the study before the pandemic officially began but finished the study after the pandemic started. The statistical significance of the main effects in all adjusted models was set at *p* < 0.05. All analyses were computed in R (version 4.2.0).

## Results

The analysis included 91 parent–child dyads who completed the baseline visit, of which 85 (93.4%) completed the month 2 visit, 83 (91.2%) completed the month 4 visit, and 79 (86.8%) completed the month 6 visit. Study participants were largely white, non-Hispanic, and of higher socioeconomic status (Table 1). The mean child emotional self-regulation CSBQ score at baseline was 4.2 (*SD* = 1.0), and the mean sleep quality CSHQ score at baseline was 38.9 (*SD* = 6.6; Table 2). No baseline measure was related to child emotional self-regulation at the *p* < 0.10 level except for household chaos (*r* = −0.28, *p* < 0.01), such that more household chaos related to poorer emotional self-regulation among children. Factors associated with worse sleep quality at baseline at the *p* < 0.10 level were younger child age (*r* = −0.25, *p* = 0.02), more total hours of screen media per week (*r* = 0.26, *p* = 0.01), a more permissive parenting style (*r* = 0.42, *p* < 0.001), and more household chaos (*r* = 0.27, *p* = 0.01).

**Table 1:** Sample characteristics at baseline.

	***N* = 91**
**Child characteristics**	
Age, mean (SD)	4.3 (0.9)
Female gender, *N* (%)	39 (42.9%)
White, non-Hispanic, *N* (%)	80 (87.9%)
WIC recipient, *N* (%)	13 (14.3%)
Total screen media, hours per week, mean (SD)	11.9 (8.3)
**Parent characteristics**	
**Age**, ***N*** **(%)**	
18–34 years	35 (38.5%)
35–39 years	41 (45.1%)
40–49 years	15 (16.5%)
Relationship to the child: mother^a^	85 (93.4%)
**Educational attainment**, ***N*** **(%)**	
Associate's degree or less	20 (22.0%)
Bachelor's degree	29 (31.9%)
Graduate or professional degree	42 (46.2%)
Married or in a domestic partnership, *N* (%)	80 (87.9%)
**Parenting style measured with the PSDQ** ^b^	
Permissive, mean (SD)	2.1 (0.5)
Authoritative, mean (SD)	4.0 (0.5)
**Household characteristics**	
**Annual household income**, ***N*** **(%)**	
< $65,0000	26 (28.6%)
$65,000–$144,999	43 (47.3%)
$145,000 or more	18 (19.8%)
Prefer not to report	4 (4.4%)
Total number of children < 18 years at the home, median (IQR)	2.0 (2.0, 3.0)
Household chaos,^c^ mean (SD)	28.2 (6.6)

Cell entries are counts with frequencies, means with standard deviations or medians with interquartile range (IQR).

^a^Mother includes biological or equivalent maternal relationships. The remaining 6 parents self-reported as fathers.

^b^PSDQ: Parenting Styles & Dimensions Questionnaire. Both subscales range from 1 to 5 with higher scores indicating higher levels of that attribute.

^c^Scores on the household chaos scale range 15–60 from with higher scores indicating more disruption and chaos in the home.

**Table 2:** Associations between child emotional self-regulation score and sleep characteristics at baseline among 3-to-5-year-olds (*n* = 91).

	**Mean (SD)**	**Correlation with emotional self-regulation**
Emotional self-regulation^a^	4.2 (1.0)	–
Nighttime sleep, hours	10.7 (1.8)	0.17 (*p* = 0.11)
Daytime napping, hours	1.6 (2.1)	**–**0.06 (*p* = 0.59)
24-h sleep, hours	12.2 (2.3)	0.08 (*p* = 0.45)
Nighttime sleep quality (CSHQ)^b^	38.9 (6.6)	**−0.24 (*****p*** **=** **0.02)**

^a^Child emotional self-regulation measured with the parent-reported Child Social Behavior Questionnaire; higher scores indicate worse emotional self-regulation (range 32–96).

^b^Child sleep quality measured with the parent-report with the Child Sleep Habits Questionnaire; higher scores indicate worse sleep quality (range 1–7).

Values in bold are statistically significant associations at the *p* < 0.05 level.

There was a small-to-medium inverse correlation between sleep quality and emotional self-regulation at baseline (*r* = −0.24, *p* = 0.02), such that worse sleep quality related to poorer emotional self-regulation (Table 2). The three measures of sleep quantity: nighttime sleep, daytime napping, and total 24-h sleep were not statistically related to child emotional self-regulation at baseline (Table 2).

The within-child difference in sleep quality measured with the CSHQ, relative to each child's mean sleep quality over the study period averaged 0.0 (as expected), with a standard deviation of 2.2 points. Child emotional self-regulation scores on the CSBQ averaged 4.3 over all children considering all study time points, with a standard deviation of 1.1. In adjusted analyses (Table 3), there was a significant within-child effect between sleep quality and emotional self-regulation. Specifically, children's emotional self-regulation was worse at study time points when their sleep quality was worse relative to their own mean sleep quality. The standardized effect size was −0.31, meaning that for each 1 standard deviation increase in a child's CSHQ score at any one time point relative to their mean CSHQ score, their score on the emotional self-regulation scale was 0.31 standard deviations lower. That translates into a decrease of 0.31 ^*^ 1.1 = 0.34 points on the CSBQ for each 2.2-point-higher score on the CSHQ scale relative to each child's mean CSHQ score.

**Table 3:** Adjusted between- and within-child associations between child change in nighttime sleep quality and emotional self-regulation over 6 months among 3-to-5-year-olds.

**Exposures**	**Outcome: Child emotional self-regulation (CSBQ), parent-reported, standardized**	
	**Among all study participants (*****n*** = **91)**	**Among those with complete data (*****n*** = **78)**^a^
	**Adjusted for household chaos**	**Adjusted for household chaos**
	β **(95% CI)**	β **(95% CI)**
**Child nighttime sleep quality (CSHQ), parent-reported, standardized**		
Between-child effect	−0.03 (−0.25, 0.18)	−0.10 (−0.32, 0.13)
Within-child effect	**−0.31 (−0.53**, **−0.09)**	**−0.35 (−0.58**, **−0.13)**
**Baseline covariates (not standardized)**		
Study visit, per 2-month increments	0.01 (−0.04, 0.05)	0.00 (−0.04, 0.05)
Child age, per year	−0.00 (−0.21, 0.21)	0.02 (−0.21, 0.25)
Child male gender	−0.23 (−0.60, 0.13)	−0.15 (−0.53, 0.23)
Permissive parenting style, PSDQ scale	−0.22 (−0.60, 0.17)	−0.25 (−0.65, 0.16)
Children's total media use, hours per week	−0.00 (−0.02, 0.02)	−0.00 (−0.03, 0.01)
Household chaos, standardized	**−0.19 (−0.35**, **−0.04)**	**−0.23 (−0.39**, **−0.07)**

Child emotional self-regulation was measured with the parent-reported Child Social Behavior Questionnaire (CSBQ); scores were reversed scored such that higher scores indicate better emotional self-regulation. Child sleep quality was measured with the parent-reported Child Sleep Habits Questionnaire (CSHQ); higher scores indicate worse sleep quality. CSBQ and CSHQ scores were standardized scores by centering at the grand sample mean. CSHQ scores were standardized before calculating the between- and within-child effects. Study visits included a baseline, month 2, month 4, and month 6 visit.

^a^Those with complete data include children with both the CSBQ and CSHQ measured at each of the four study visits.

Values in bold are statistically significant associations at the *p* < 0.05 level.

The between-child main effects were not statistically different from zero, and the only covariate statistically associated with emotional self-regulation was household chaos measured at baseline. That standardized effect size was −0.19, meaning that children's scores on the emotional self-regulation scale, on average, were −0.19 standard deviations lower for each 1 standard deviation increase in household chaos. That translates into a decrease, on average, of 0.19 ^*^ 1.1 = 0.21 points on the CSBQ for each 6.6-point increase on the household chaos scale. All findings were consistent in the subset of children with data at all four study timepoints, with the within-child effects slightly larger in that subset (Table 2). Findings were consistent in all sensitivity analyses when adjusting for whether the study visit occurred before or after the start of the pandemic.

## Discussion

In this study, it was found that worse sleep quality, as measured by the CSHQ, was associated with worse emotional self-regulation, as measured by CSBQ, in children aged 3–5 years. This relationship remained when analyzing within-child changes over 6 months, and we observed that if sleep quality was worse at any one time point, emotional self-regulation was worse at that time point too. Understanding the relationship in this age group is important because poor sleep and emotional self-regulation in childhood can have negative outcomes on behavior and health in later life (Gruber et al., 2014; Chaput et al., 2017).

We also evaluated covariates that may relate to both sleep and emotional self-regulation. In our study, however, the association between worse sleep quality and emotional self-regulation was maintained even when adjusting for covariates, including child age, sex, permissive parenting style, children's total media use, and household chaos, which supports that sleep quality may indeed have a direct, causal impact on children's emotional self-regulation. Two of those modifiable covariates—children's total media use and permissive parenting style—may be causally related to poor sleep among children (Chaput et al., 2017; LeBourgeois et al., 2017; Shetty et al., 2022). Thus, it is possible that a non-significant effect in the adjusted model between those covariates and children's emotional self-regulation reflects a mediating effect of poor sleep quality between those factors and children's emotional regulation. This current analysis did, however, find that greater household chaos was independently related to worse child emotional self-regulation, even when adjusted for sleep quality, a finding that aligns with other studies demonstrating a relationship between greater household chaos and worse child outcomes (Marsh and Dobson, 2020). For example, household chaos has been shown to be associated with a number of adverse outcomes for children, such as behavioral, attention, sleep, and other health problems (Emond, 2020; Marsh and Dobson, 2020). Whether household chaos may also partially mediate a link between poor sleep and children's emotional self-regulation is unclear; household chaos was measured at the baseline visit, and additional studies are needed that can precisely measure such temporal relationships.

Interestingly, we found that sleep duration (total 24-h sleep, daytime naps, or total nighttime sleep) was not significantly associated with children's emotional self-regulation. One systematic review concluded that decreased sleep duration has been linked to worse emotional regulation in children aged 0–4 years as demonstrated by two randomized control trials and two longitudinal studies (Chaput et al., 2017). The other three longitudinal studies in the systematic review, however, reported no relationship between sleep and emotion regulation (Chaput et al., 2017). Together, our findings and previous findings indicate the importance of not only focusing on sleep duration but sleep quality as well.

Our study has several limitations worth noting. While measuring sleep quality and emotional self-regulation in young children can be difficult, we chose validated questionnaires that would give us quantitative measurements. The CSBQ and the CSHQ, however, were originally designed for a different population than ours and then were adopted for healthy younger children like those in our study. While other studies have demonstrated good reliability of these surveys in various populations, it remains a limitation. Additionally, these surveys were answered by the children's parents, which may not always reflect the child's viewpoint. This method of reporting, however, is likely appropriate in this age group, and using these validated questionnaires allows for easier reproducibility in future studies. The data were collected at two time points, including baseline and month 6, which may not be sufficient to fully understand the trends of sleep quality, sleep duration, and emotional regulation. Although there was a correlation between worse sleep quality and worse emotional self-regulation, the effect size was relatively small and may not represent a clinically significant outcome. However, small effects may compound and become large problems with age if sleep quality is not corrected. Importantly, this sample represented young (3–5 years old), mostly white children living in homes with highly educated married parents; therefore, the results of this study may not be generalizable to other populations.

In conclusion, a within-child effect was found between poorer sleep quality and worse emotional self-regulation among 3- to 5-year-old children over a 6-month period. That finding was adjusted for a between-child effect, suggesting that the within-child effect may be causal. The limited heterogeneity among the sampled population reduces generalizability. Regardless, findings contribute to the understanding of how sleep may impact emotional self-regulation at a young age.

## Data availability statement

The original contributions presented in the study are included in the article/supplementary material, further inquiries can be directed to the corresponding author.

## Ethics statement

The studies involving humans were approved by Dartmouth's Committee for the Protection of Human Subjects. The studies were conducted in accordance with the local legislation and institutional requirements. Written informed consent for participation in this study was provided by the participants' legal guardians/next of kin. Written informed consent was obtained from the minor(s)' legal guardian/next of kin for the publication of any potentially identifiable images or data included in this article.

## Author contributions

CG: Writing – review & editing, Writing – original draft. DC: Project administration, Writing – review & editing. OP: Writing – review & editing. GB: Writing – review & editing. JE: Conceptualization, Funding acquisition, Formal analysis, Writing – review & editing, Writing – original draft.
